# Diabetes and Cardiovascular Disease Risk Perception and Risk Indicators: a 5-Year Follow-up

**DOI:** 10.1007/s12529-020-09924-2

**Published:** 2020-08-17

**Authors:** Marleena Vornanen, Hanna Konttinen, Markku Peltonen, Ari Haukkala

**Affiliations:** 1 Department of Social Research, University of Helsinki, Unioninkatu 37, PL 54, 00014 Helsinki, Finland; 2 Department of Food and Nutrition, University of Helsinki, Helsinki, Finland; 3 National Institute for Health and Welfare, Helsinki, Finland

**Keywords:** Type 2 diabetes, Cardiovascular disease, Risk perception, Self-efficacy, Lifestyle factors, Longitudinal

## Abstract

**Background:**

Perceived disease risk may reflect actual risk indicators and/or motivation to change lifestyle. Yet, few longitudinal studies have assessed how perceived risk relates to risk indicators among different disease risk groups. We examined in a 5-year follow-up, whether perceived risks of diabetes and cardiovascular disease predicted physical activity, body mass index (BMI kg/m^2^), and blood glucose level, or the reverse. We examined further whether perceived risk, self-efficacy, and outcome beliefs together predicted changes in these risk indicators.

**Method:**

Participants were high diabetes risk participants (*N* = 432) and low/moderate-risk participants (*N* = 477) from the national FINRISK 2002 study who were followed up in 2007. Both study phases included questionnaires and health examinations with individual feedback letters. Data were analyzed using gender- and age-adjusted structural equation models.

**Results:**

In cross-lagged autoregressive models, perceived risks were not found to predict 5-year changes in physical activity, BMI, or 2-h glucose. In contrast, higher BMI and 2-h glucose predicted 5-year increases in perceived risks (*β*-values 0.07–0.15, *P*-values < 0.001–0.138). These associations were similar among high- and low/moderate-risk samples. In further structural equation models, higher self-efficacy predicted increased physical activity among both samples (*β*-values 0.10–0.16, *P*-values 0.005–0.034). Higher outcome beliefs predicted lower BMI among the low/moderate-risk sample (*β*-values − 0.04 to − 0.05, *P*-values 0.008–0.011).

**Conclusion:**

Perceived risk of chronic disease rather follows risk indicators than predicts long-term lifestyle changes. To promote sustained lifestyle changes, future intervention studies need to examine the best ways to combine risk feedback with efficient behavior change techniques.

## Introduction

Type 2 diabetes mellitus and cardiovascular diseases (CVD) cause a significant disease burden worldwide [1]. In 2015, approximately 8.8% of adults worldwide had diabetes, and the proportion is expected to rise to 10.4% by 2040 [2]. Sustained changes in weight, fat and fiber intake, and physical activity reduce the risk of illness [3]. Many health behavior theories [4] include risk perception as a predictor of preventive intentions and behavior. Also, healthcare professionals and the media often remind people that, for example, obesity and lack of physical activity are risk factors for chronic diseases.

The aim of risk communication is to promote (1) *accurate* risk perceptions and (2) *motivation* to change health behavior to prevent illness. Hence, the association between risk indicators and perceived risk is assumed to be bidirectional. Perceived risk is expected to motivate preventive behavior (behavioral motivation hypothesis), but people are also supposed to adjust their risk perception to match their current risk indicators (accuracy hypothesis) [5]. Risk communication may promote more accurate risk perceptions [6]. Also, longitudinal studies suggest that, after behavior change, people re-adjust their risk perceptions [7]. Randomized controlled trials show that feedback on physiological risk indicators may motivate health behavior change [8], but a recent review concludes that personalized risk feedback alone tends not to result in sustained health behavior change [9]. As personalized medicine promotes individualized risk assessment and treatment in healthcare [10], research is needed on whether risk perception relates to health behavior similarly among those with a high disease risk and those with a lower risk.

Perceived risk of disease is usually not enough to succeed in effortful lifestyle changes, such as weight loss or maintaining regular physical activity [11]. Knowledge and risk perception alone do not guarantee intention to change lifestyle [12]. According to several health behavior theories, for instance the health action process approach (HAPA) [13], intention to change health behavior requires self-efficacy and positive outcome beliefs. Self-efficacy means feeling capable of making the lifestyle change. Outcome beliefs mean believing that the lifestyle change will prevent disease effectively. For example, if one perceives high risk of chronic disease, and one feels capable of increasing physical activity (self-efficacy), and one believes that physical activity will prevent the illness (outcome beliefs), one is expected to form an intention to increase their physical activity. Then, translating intention into long-term action requires self-efficacy to maintain the physical activity, and to re-adopt it after relapse.

A meta-analysis by Gholami et al. [14] included 11—mostly longitudinal—studies that used the HAPA model to predict physical activity. Risk perception did not promote intention to be physically active, but self-efficacy and outcome beliefs did. Intention had an effect on actual physical activity [14]. Other reviews of experimental [15, 16] and correlational [16] studies showed that risk perception had a small effect on health behavior intentions and health behavior in general. In the end, successful health behavior change may change physiological risk indicators, such as body weight and blood glucose. Yet, to our knowledge, no longitudinal studies have simultaneously explored associations between perceived risks of chronic diseases, self-efficacy, outcome beliefs, and changes in preventive health behavior and physiological risk indicators.

The aim of this study was to examine the longitudinal association of perceived risk and risk indicators, in a 5-year follow-up. The main research question was whether perceived risk of diabetes or CVD predicted changes in physical activity, body mass index (BMI kg/m^2^), or blood glucose level, or whether the risk indicators rather predicted changes in perceived risks. Furthermore, the study examined how perceived risk of diabetes or CVD, self-efficacy, and outcome beliefs together predicted changes in physical activity, BMI, and blood glucose. The direction of the longitudinal associations between perceived risk and risk indicators remained an open research question. Higher self-efficacy and outcome beliefs were hypothesized to predict more frequent physical activity, lower BMI, and lower blood glucose over 5 years. The analyses were performed among two samples of people with a different diabetes risk status.

## Methods

### Participants

**Fig. 1:**
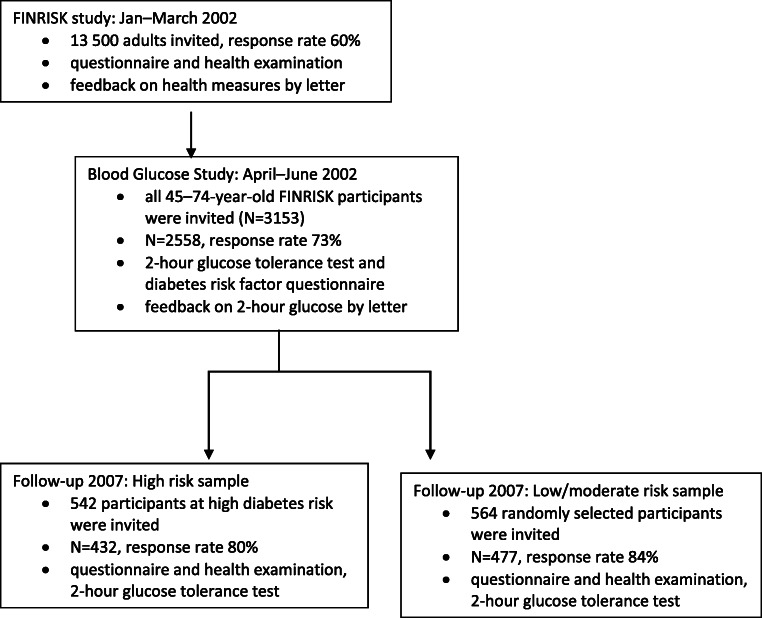
Flow of participants and data collection process in the FINRISK 2002 study and the FINRISK Blood Glucose Study

Participants (Fig. 1) were originally from the national FINRISK 2002 study (Jan–Apr) [17], conducted by the Finnish National Institute for Health and Welfare. In the FINRISK 2002, a health survey and an invitation to a health examination were mailed to a random, population register–based, sample of 13,500 people. The sample was stratified by gender, 10-year age groups, and six geographical regions. Participants responded to the health survey at home, and returned it when they attended the health examination in a municipal healthcare center. Participants received feedback on several biomarkers (including cholesterol, blood pressure, BMI, waist circumference) via letter. The letter stated the normal range for each measure, the participant’s personal test result, and how to act if the result exceeded the normal range, e.g. change their diet, lose weight, or contact their personal doctor if the value was very high.

All men and women aged 45–74 years who participated (*N* = 3513) in the FINRISK 2002 were later invited to the FINRISK Blood Glucose Study (Apr–June 2002), which included a glucose tolerance test and another questionnaire [18] related to diabetes risk indicators. Afterwards, the participants (*N* = 2558, participation rate 73%) received a feedback letter that reported their fasting plasma glucose, 2-h plasma glucose, and insulin level. Recommended levels for fasting glucose (< 6.0 mmol/l) and 2-h glucose (< 7.8 mmol/l) were stated. Participants were recommended to have their blood glucose re-measured if either of the values exceeded the recommended level, or to contact their personal doctor if their fasting glucose was over 7.0 mmol/l and/or their 2-h glucose was over 11.1 mmol/l (diagnostic criteria for diabetes, this was not stated in the letter). The letter also said that slightly elevated blood glucose could be treated by increasing exercise and fiber intake, decreasing fat intake, or achieving a normal weight.

All participants with a high diabetes risk (*N* = 432, participation rate 80%) and a randomly selected sample of participants with a low or moderate risk (*N* = 477, participation rate 84%) of the Blood Glucose Study were invited to attend a follow-up study 5 years later in 2007. Because of this sampling protocol, all statistical analyses of the current study were performed separately among the high-risk sample and the low/moderate-risk sample. A participant was classified to be at high diabetes risk if they filled one or more of the following criteria at baseline: (1) elevated fasting or 2-h glucose [19], (2) high diabetes risk score based on the questionnaire (> 14 points) [18], or (3) self-reported current or previous CVD. The follow-up invitation letter did not state whether the participant was classified to be at high risk for diabetes. At follow-up in 2007, the participants filled in another survey and attended a health examination, which included another glucose tolerance test.

### Measures

Perceived absolute lifetime risk of diabetes was measured at baseline and at follow-up, before health examinations, with a single item: How do you evaluate your own risk of getting diabetes in your lifetime? 0 = I have diabetes, 1 = very low, 2 = low, 3 = moderate, 4 = high, 5 = very high. In a previous study, a similar 5-point scale correlated highly with a more continuous measure of perceived absolute risk, and moderately with perceived comparative risk [20]. Before the baseline health examination, none in the low/moderate-risk sample and one participant (*N* = 1) in the high-risk sample reported having diabetes. At follow-up before the health examination, diabetes was reported by two participants (*N* = 2) in the low/moderate-risk sample, and by 38 participants (*N* = 38) in the high-risk sample. Those who responded “0 = I have diabetes” at baseline or at follow-up were excluded from the analyses where the relevant measure was used. As a result, a 5-point scale for perceived diabetes risk was used in the analyses.

Perceived absolute lifetime risk of CVD was assessed with a similar measure as perceived risk of diabetes (above). Similarly, those who self-reported having CVD were excluded from the analyses where the measure was used (at baseline *N* = 20 in the low/moderate diabetes risk sample, and *N* = 35 in the high-risk sample; at follow-up *N* = 29, and *N* = 36, respectively). A 5-point scale for perceived CVD risk was used in the analyses.

Health action self-efficacy was measured at baseline with 6 items (scale from 1 = very uncertain to 4 = very certain). How certain are you that… (1) you will be able to take the health perspective into account when planning your life and making decisions on it? (2) you will manage to follow your decisions to start a new, healthier life? (3) you will manage to follow a healthy lifestyle, even if people around you did not care about it? (4) you will be able to resist temptations when you know they harm your health? (5) you will manage to care whether something is harmful for your health or not, even if you were busy, tired, or under a lot of pressure? (6) you will be able to take the health perspective into account, even if it was inconvenient or you had to give up other things that are important to you? For the descriptive and correlational analyses, a mean score of the 6 self-efficacy items was calculated. In all other analyses, self-efficacy was modeled as a latent variable.

Outcome beliefs were measured with a single item (scale from 1 = very uncertain to 4 = very certain): How certain are you that serious illnesses, such as heart diseases, cancer or diabetes, can be prevented by healthy lifestyle?

Frequency of weekly physical activity was assessed with a single open-ended question at baseline: How many times a week, in your free time, do you exercise so that you experience at least mild exhaustion and sweating? At follow-up, the question further specified that each physical activity time had to take at least 20 min, and offered response choices: 1 = I cannot exercise due to illness or injury, 2 = less than once a week, 3 = once a week … 7 = five times a week or more. For the analyses, responses to these baseline and follow-up physical activity questions were recoded into the same scale: 0 = less than once a week, 1 = once a week, 2 = twice a week, 3 = three times a week, 4 = four times a week, 5 = five times a week or more. Those who reported incapability to exercise at follow-up were excluded from the analyses on physical activity (low/moderate-risk sample *N* = 13, high-risk sample *N* = 38).

Body mass index (BMI) was calculated as weight (kg) divided by squared height (m) [21], which were measured by trained research nurses in the health examinations at baseline and at follow-up.

Two-hour plasma glucose was measured in the health examinations at baseline and at follow-up according to WHO guidelines [19]. After a 10-h fast, the participants drank 300-ml solution that contained 75 g anhydrous glucose and 1.6 g citric acid. After 2 h, a blood sample was drawn to measure their plasma glucose level [22]. We chose to use 2-h plasma glucose as the outcome measure, since it is more sensitive in predicting progress to diabetes, compared with fasting plasma glucose [23, 24].

### Statistical Analyses

Structural equation modeling with a maximum likelihood estimator was used as the main analytical approach, using SPSS Amos Graphics version 24 software (IBM Corp., Armonk, NY, USA). This method was chosen as it allows the use of cross-lagged autoregressive models and latent variables. Descriptive analyses were conducted using SPSS Statistics version 24 (IBM Corp., Armonk, NY, USA). Differences in the prevalences and means were tested between the high-risk sample and the low/moderate-risk sample: *χ*^2^ test was used for dichotomous variables (effect size estimate phi) and one-way ANOVA for other variables (effect size estimate partial eta^2^). All structural equation models were tested separately for the high diabetes risk sample and the low/moderate-risk sample, and adjusted for gender and age.

**Fig. 2:**
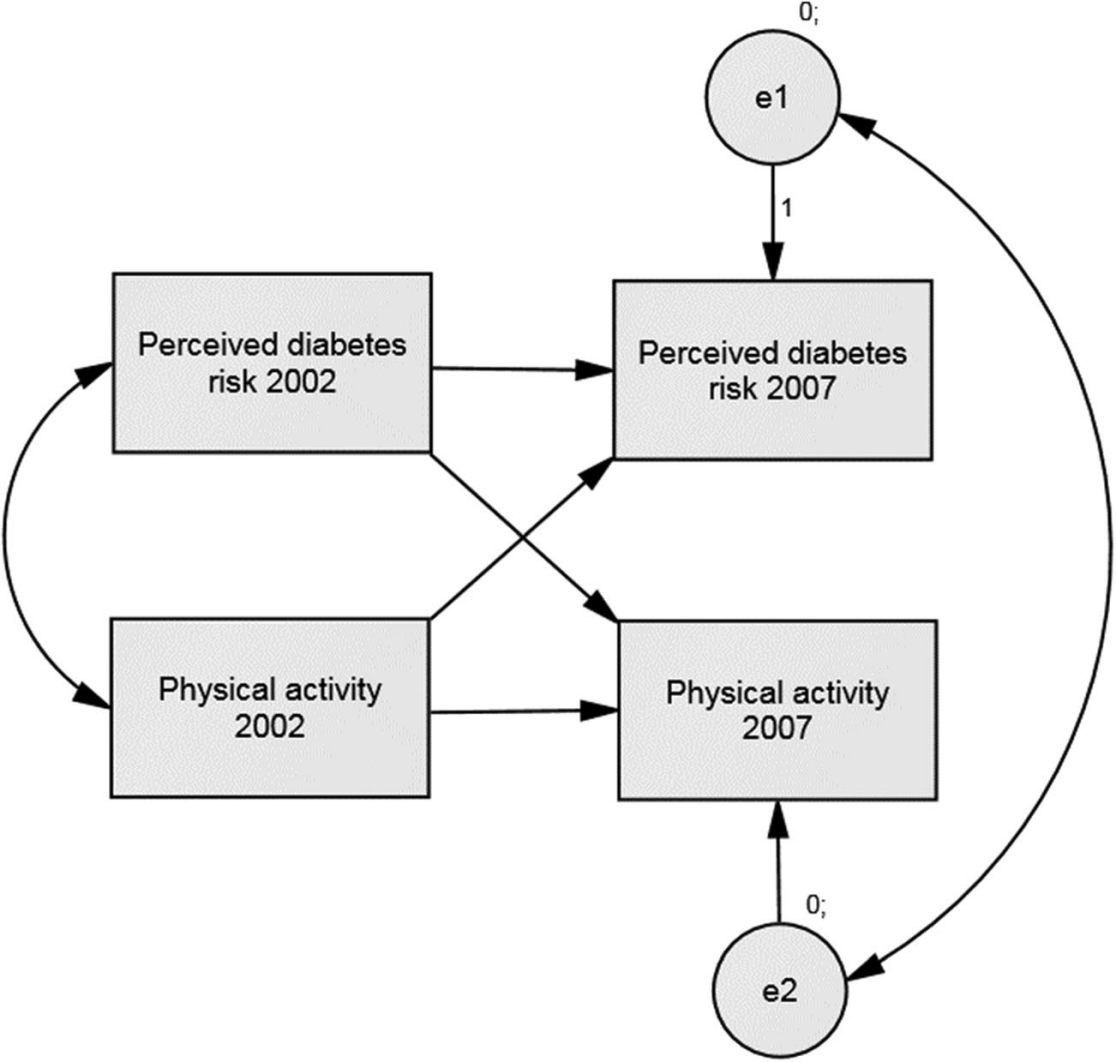
Example of a cross-lagged autoregressive model between perceived risk of disease and risk indicator (adjusted for age and gender)

Cross-lagged autoregressive models were used to examine the longitudinal associations between perceived risk and risk factors. Model specification is presented in Fig. 2. We tested whether perceived risk of diabetes in 2002 predicted physical activity, BMI, or 2-h glucose in 2007, or whether the risk indicators in 2002 rather predicted perceived diabetes risk in 2007. Similarly, we tested whether perceived risk of CVD predicted physical activity or BMI, or the reverse. Fit of the cross-lagged autoregressive models was not evaluated, since they were saturated models (i.e., just-identified with zero degrees of freedom). Multigroup analyses were conducted, to test whether the cross-lagged associations between perceived risks and risk indicators were similar among the low/moderate-risk sample and the high-risk sample. Each cross-lagged regression path was tested separately (altogether 10 tests): the path was constrained to be similar among both samples, and the fit of this constrained model was compared with the fit of the saturated unconstrained model using the *χ*^2^ difference test.

We tested structural equation models that predicted physical activity, BMI, or 2-h glucose in 2007. The main predictors were baseline perceived diabetes risk, self-efficacy, and outcome beliefs. We first tested models with each main predictor separately and then conducted multivariate models that included all three main predictors simultaneously (all models were adjusted for gender, age, and the outcome variable level in 2002). We tested similar models that predicted physical activity or BMI, using perceived CVD risk, self-efficacy, and outcome beliefs as the main predictors. The fit of each model was evaluated with several fit indexes: *χ*^2^ statistic, Comparative Fit Index (CFI), and root mean square error of approximation (RMSEA) [25].

Before analyzing structural equation models where self-efficacy was included, confirmatory factor analysis was used to test the one-factor structure of the self-efficacy measure. All 6 items had moderate or high factor loadings on the self-efficacy latent factor among the low/moderate-risk sample (*λ* = 0.54–0.78, all *P*-values < 0.001) and among the high-risk sample (*λ* = 0.56–0.80, all *P*-values < 0.001). Of the fit indexes, particularly CFI suggested that the one-factor model had a reasonable fit with the data (*χ*^2^ = 94.97, df = 9, *P* < 0.001; CFI = 0.934; RMSEA = 0.142 for the low/moderate-risk sample and *χ*^2^ = 65.15, df = 9, *P* < 0.001; CFI = 0.953; RMSEA = 0.120 for the high-risk sample) [25].

## Results

**Fig. 3:**
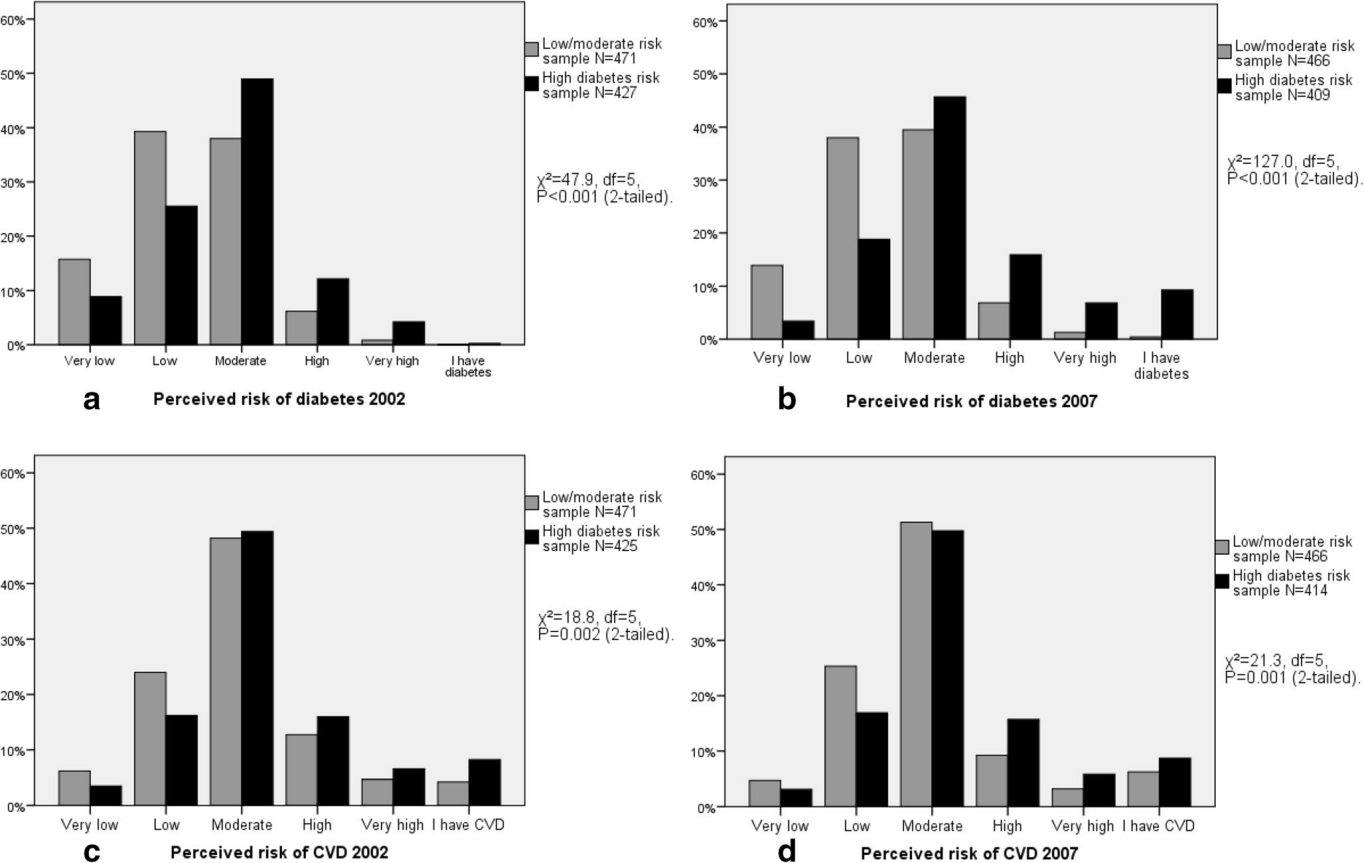
**a**–**d** Distributions of perceived lifetime risks of diabetes and CVD before health examinations at baseline in 2002 and at follow-up in 2007

Descriptive characteristics of the study participants at baseline and at follow-up are presented in Table 1. At both measurement points, before the health examinations, most participants perceived their disease risks low or moderate, although risk perceptions were higher among the high diabetes risk sample (Fig. 3, Table 1). At baseline, mean levels of self-efficacy and outcome beliefs were moderate among both samples (close to value 3 = quite certain). Obesity, high blood glucose, and family history of diabetes were more common among the high diabetes risk sample, whereas no difference was found in the self-reported frequency of physical activity (Table 1). At follow-up, before the health examination, self-reported prevalence of diabetes was 0.4% in the low/moderate-risk sample (for CVD 6.1%) and 8.8% in the high-risk sample (for CVD 8.3%). At follow-up health examination, however, a third of the high-risk sample were diagnosed with diabetes (Table 1). Bivariate correlations between the primary study variables are presented in Table 2. In both samples, perceived disease risks were consistently associated with higher BMI, whereas perceived risks showed weaker associations with physical activity and 2-h glucose. Participants with a higher self-efficacy had lower BMI and reported more frequent physical activity.

**Table 1:** Descriptive characteristics of the study samples (FINRISK Blood Glucose Study 2002)

Baseline 2002	Low/moderate-risksample, *N* = 451–477	High diabetes risksample, *N* = 390–432	Min–max	Group difference*P* value	Partial eta^2^/phi
Women (%)	57.7	56.9		.829	− .01
Age mean (SD)	55.9 (6.9)	59.3 (7.0)	45–74	< .001	.06
Body mass index (kg/m^2^) mean (SD)	27.1 (4.2)	30.3 (4.7)	18.8–45.6	< .001	.12
Body mass index ≥ 30 (%)	21.0	47.0		< .001	.28
Physical activity mean (SD)	2.3 (1.6)	2.2 (1.7)	0–5	.215	.00
2-h glucose mean (SD)	5.8 (1.1)	9.2 (3.1)	0.7–29.5	< .001	.36
Perceived diabetes risk mean (SD)	2.4 (0.9)	2.8 (0.9)	1–5	< .001	.05
Perceived cardiovascular disease risk mean (SD)	2.9 (0.9)	3.1 (0.9)	1–5	.001	.01
Family history of diabetes (%)^a^	33.1	50.2		< .001	.17
Family history of early myocardial infarction (%)^b^	29.4	35.6		.043	.07
Self-efficacy mean (SD)^c^	2.8 (0.5)	2.7 (0.6)	1–4	.039	.01
Outcome beliefs mean (SD)	3.1 (0.7)	3.1 (0.7)	1–4	.823	.00
Follow-up 2007	*N* = 437–477	*N* = 416–432			
Women (%)	57.7	56.9		0.829	− .01
Body mass index mean (SD)	27.0 (4.5)	30.2 (5.0)	17.7–48.3	< 0.001	.10
Body mass index ≥30 (%)	21.9	47.1		< 0.001	.27
Physical activity mean (SD)	2.6 (1.5)	2.4 (1.6)	0–5	0.145	.00
2-h glucose mean (SD)	6.3 (1.9)	8.7 (3.0)	2.4–25.8	< 0.001	.18
Perceived diabetes risk mean (SD)	2.4 (0.9)	3.0 (0.9)	1–5	< 0.001	.11
Perceived cardiovascular disease risk mean (SD)	2.8 (0.8)	3.0 (0.9)	1–5	< 0.001	.02
Glucose-lowering medication (%)	0.6	11.1		0.024	.18
Onset of diabetes (%)	6.4	34.2		< 0.001	.35

^a^One or more first-degree relatives diagnosed with diabetes

^b^One or more first-degree relatives with a myocardial infarction before the age of 60

^c^Mean score of 6 items (scale 1–4)

Group differences were tested through the *χ*^2^ test (dichotomous variables, effect size estimate phi) or one-way ANOVA (effect size estimate partial eta^2^)

**Table 2:** Bivariate correlations between the primary study variables (FINRISK Blood Glucose Study 2002)

**Low/moderate diabetes risk sample (cases excluded pairwise,** ***N*** **= 418–477)**											
	**1**	**2**	**3**	**4**	**5**	**6**	**7**	**8**	**9**	**10**	**11**
1 Perceived diabetes risk 2002											
2 Perceived diabetes risk 2007	.58***										
3 Perceived CVD risk 2002	.42***	.37***									
4 Perceived CVD risk 2007	.29***	.42***	.64***								
5 Self-efficacy (sum variable of 6 items)	− .21***	− .19***	− .24***	− .13**							
6 Outcome beliefs	− .10*	− .11*	− .07	− .04	.19***						
7 Physical activity 2002	− .06	.04	− .11*	− .10*	.30***	.07					
8 Physical activity 2007	.00	− .05	− .10*	− .09	.26***	.03	.52***				
9 Body mass index 2002	.29***	.28***	.14**	.18***	− .17**	− .01	− .01	.01			
10 Body mass index 2007	.29***	.32***	.13**	.20***	− .17***	− .04	.01	− .04	.93***		
11 2-h glucose 2002	− .02	.09	.03	.04	− .01	− .06	.02	.06	.17***	.14**	
12 2-h glucose 2007	.05	.17***	.07	.09	− .02	− .05	.11*	− .00	.19***	.19***	.31***
**High diabetes risk sample (cases excluded pairwise,** ***N*** **= 315–432)**											
	**1**	**2**	**3**	**4**	**5**	**6**	**7**	**8**	**9**	**10**	**11**
1 Perceived diabetes risk 2002											
2 Perceived diabetes risk 2007	.55***										
3 Perceived CVD risk 2002	.38***	.26***									
4 Perceived CVD risk 2007	.27***	.37***	.59***								
5 Self-efficacy^a^	− .15**	− .10	− .19***	− .19***							
6 Outcome beliefs	.01	.05	− .08	− .07	.27***						
7 Physical activity 2002	− .08	− .07	− .15**	− .14**	.36***	.10*					
8 Physical activity 2007	− .04	− .06	− .06	− .12*	.27***	.02	.43***				
9 Body mass index 2002	.23***	.18***	.15**	.18***	− .15**	.05	− .13*	− .18**			
10 Body mass index 2007	.20***	.20***	.12*	.20***	− .12*	.06	− .08	− .23***	.90***		
11 2-h glucose 2002	.13**	.16**	.04	.01	− .02	− .04	− .06	− .07	.08	.04	
12 2-h glucose 2007	.04	.13*	.05	.07	− .07	− .02	− .12*	− .11	.15**	.19***	.48***

^a^Mean score of 6 items (scale 1–4)

*P < .05; **P < .01; ***P < .001 (2-tailed)

Figure 2 presents an example of the tested cross-lagged autoregressive models, whereas Table 3 presents individual path regression coefficients from the models. Baseline perceived risk of diabetes was not found to predict physical activity (Table 3A), BMI (Table 3B), or 2-h glucose (Table 3C) 5 years later in either sample. The reverse associations were found: higher perceived risk of diabetes in 2007 was predicted by higher physical activity (low/moderate-risk sample *β =* 0.08, *P* = 0.035), BMI (low/moderate-risk sample *β* = 0.15, *P* < 0.001), and 2-h glucose (high-risk sample *β* = 0.13, *P* = 0.014) in 2002.

**Table 3:** Results from age- and gender-adjusted cross-lagged autoregressive models (FINRISK Blood Glucose Study 2002)

A. Perceived diabetes risk—physical activity		*B*	Std. *B*	SE *B*	*P* value	D. Perceived CVD risk—physical activity		*B*	Std. *B*	SE *B*	*P* value
Low/moderate risk, *N* = 462	PR02→PR07	.60	.58	.04	< .001	Low/moderate risk, *N* = 431	PR02→PR07	.59	.64	.04	< .001
	PA02→PA07	.50	.51	.04	< .001		PA02→PA07	.52	.52	.04	< .001
	PA02→PR07	.04	.08	.02	.035		PA02→PR07	.00	.00	.02	.978
	PR02→PA07	.07	.04	.08	.339		PR02→PA07	− .08	− .05	.07	.264
High diabetes risk, *N* = 360	PR02→PR07	.54	.53	.05	< .001	High diabetes risk, *N* = 352	PR02→PR07	.59	.59	.04	< .001
	PA02→PA07	.41	.42	.05	< .001		PA02→PA07	.40	.41	.05	< .001
	PA02→PR07	− .00	− .00	.03	.965		PA02→PR07	− .02	− .05	.02	.322
	PR02→PA07	.01	.01	.09	.911		PR02→PA07	.00	.00	.09	.986
B. Perceived diabetes risk—body mass index		*B*	Std. *B*	SE *B*	*P* value	E. Perceived CVD risk—body mass index		*B*	Std. *B*	SE *B*	*P* value
Low/moderate risk, *N* = 475	PR02→PR07	.55	.54	.04	< .001	Low/moderate risk, *N* = 439	PR02→PR07	.57	.62	.03	< .001
	BMI02→BMI07	1.00	.94	.02	< .001		BMI02→BMI07	1.01	.94	.02	< .001
	BMI02→PR07	.03	.15	.01	< .001		BMI02→PR07	.02	.10	.01	.006
	PR02→BMI07	.03	.01	.09	.710		PR02→BMI07	− .03	−. 01	.09	.744
High diabetes risk, *N* = 393	PR02→PR07	.53	.52	.05	< .001	High diabetes risk sample, *N* = 380	PR02→PR07	.56	.57	.04	< .001
	BMI02→BMI07	.93	.90	.02	< .001		BMI02→BMI07	.93	.90	.02	< .001
	BMI02→PR07	.01	.07	.01	.138		BMI02→PR07	.02	.09	.01	.037
	PR02→BMI07	− .09	− .02	.12	.478		PR02→BMI07	− .10	− .02	.13	.466
C. Perceived diabetes risk—2-h glucose		*B*	Std. *B*	SE *B*	*P* value						
Low/moderate risk, *N* = 475	PR02→PR07	.74	.73	.10	< .001						
	GL02→GL07	.45	.27	.08	< .001						
	GL02→PR07	.11	.14	.06	.058						
	PR02→GL07	.14	.06	.21	.500						
High diabetes risk, *N* = 393	PR02→PR07	.51	.50	.05	< .001						
	GL02→GL07	.63	.51	.07	< .001						
	GL02→PR07	.05	.13	.02	.014						
	PR02→GL07	.14	.04	.17	.399						

*CVD*, cardiovascular disease; *PR02*, perceived risk 2002; *PR07*, perceived risk 2007; *PA02*, physical activity 2002; *PA07*, physical activity 2007; *BMI02*, body mass index 2002; *BMI07*, body mass index 2007; *GL02*, 2-h glucose 2002; *GL07*, 2-h glucose 2007

In similar models on perceived CVD risk, baseline perceived risk of CVD was not found to predict physical activity or BMI (Table 3D and E) in either sample. The reverse association was found for BMI but not for physical activity: higher BMI predicted higher perceived risk of CVD among the low/moderate-risk sample (*β* = 0.10, *P* = 0.006) and among the high-risk sample (*β* = 0.09, *P* = 0.037). In any of the cross-lagged associations, there were no statistically significant differences between the low/moderate-risk sample and the high-risk sample (∆*χ*^2^ values 0.10–2.21, ∆df = 1, *P*-values 0.137–0.752).

Multivariate structural equation models that predicted physical activity, BMI, or 2-h glucose in 2007 (adjusted for gender, age, and the outcome variable level in 2002) showed that among both samples, those with a higher baseline self-efficacy were more physically active 5 years later (Table 4A and D), but self-efficacy was not found to predict BMI or 2-h glucose. Among the low/moderate-risk sample, those with higher baseline outcome beliefs had slightly lower BMI at follow-up (Table 4B and E), but outcome beliefs were not found to predict physical activity or 2-h glucose. Perceived risk of diabetes or CVD was not found to predict physical activity, BMI, or 2-h glucose in either sample. Results from the models that included each main predictor separately (adjusted for gender, age, and the outcome variable level in 2002) were highly similar as the multivariate results (results not shown).

**Table 4:** Results from structural equation models predicting 5-year changes in physical activity, body mass index, and 2-h glucose (FINRISK Blood Glucose Study 2002)

	Low/average-risk sample					High diabetes risk sample				
	*B*	Std. *B*	SE *B*	*P* value	*r*^2^	*B*	Std. *B*	SE *B*	*P* value	*r*^2^
A. Dependent variable: physical activity 2007^a^	*N* = 464					*N* = 393				
Perceived diabetes risk 2002	.10	.05	.08	.208	.29	.01	.00	.08	.934	.22
Self-efficacy 2002	.38	.13	.14	.005		.49	.16	.18	.005	
Outcome beliefs 2002	− .05	− .02	.10	.615		− .11	− .05	.11	.328	
B. Dependent variable: body mass index 2007^b^	*N* = 477					*N* = 431				
Perceived diabetes risk 2002	.02	.00	.09	.807	.88	− .05	− .01	.12	.680	.82
Self-efficacy 2002	− .01	.02	.16	.790		.26	.03	.23	.246	
Outcome beliefs 2002	−. 29	− .04	.12	.011		.01	.00	.16	.970	
C. Dependent variable: 2-h glucose 2007^c^	*N* = 477					*N* = 431				
Perceived diabetes risk 2002	.12	.06	.10	.218	.13	.20	.06	.15	.174	.40
Self-efficacy 2002	− .07	− .02	.17	.682		− .19	− .03	.29	.524	
Outcome beliefs 2002	− .03	− .02	.13	.787		.05	.01	.20	.824	
D. Dependent variable: physical activity 2007^d^	*N* = 448					*N* = 365				
Perceived cardiovascular disease risk 2002	− .05	− .03	.07	.471	.30	.02	.02	.09	.786	.22
Self-efficacy 2002	.28	.10	.13	.034		.48	.16	.18	.009	
Outcome beliefs 2002	− .04	− .02	.10	.662		− .11	− .05	.12	.366	
E. Dependent variable: body mass index 2007^e^	*N* = 457					*N* = 397				
Perceived cardiovascular disease risk 2002	− .03	− .01	.09	.720	.88	− .08	− .02	.13	.548	.81
Self-efficacy 2002	− .05	− .01	.16	.770		.19	.02	.24	.421	
Outcome beliefs 2002	− .32	− .05	.12	.008		.04	.01	.17	.819	

Self-efficacy (6 items) was modeled as a latent variable

^a^Adjusted for age, gender, and physical activity 2002. Model fit for the whole data: *χ*^2^ = 289.53, df = 78, *P* < 0.001; CFI = 0.926; RMSEA = 0.056

^b^Adjusted for gender, age, and body mass index 2002. Model fit for the whole data: *χ*^2^ = 296.93, df = 78, *P* < 0.001; CFI = 0.951; RMSEA = 0.056

^c^Adjusted for gender, age, and 2-h glucose 2002. Model fit for the whole data: *χ*^2^ = 281.45, df = 78, *P* < 0.001; CFI = 0.928; RMSEA = 0.054

^d^Adjusted for gender, age, and physical activity 2002. Model fit for the whole data: *χ*^2^ = 282.64, df = 78, *P* < 0.001; CFI = 0.926; RMSEA = 0.057

^e^Adjusted for gender, age, and body mass index 2002. Model fit for the whole data: *χ*^2^ = 292.86, df = 78, *P* < 0.001; CFI = 0.949; RMSEA = .057

*CFI*, Comparative Fit Index, *RMSEA*, root mean square error of approximation

Since the use of glucose-lowering medication could affect the association between perceived diabetes risk and 2-h glucose, we performed sensitivity analyses. Those who self-reported the use of glucose-lowering medication were excluded from the cross-lagged model on 2-h glucose (low/moderate-risk sample *N* = 1, high-risk sample *N* = 15). The results remained relatively similar to Table 3C: perceived risk was not found to predict 2-h glucose, but 2-h glucose predicted perceived risk (low/moderate-risk sample *β* = 0.11, *P* = 0.006; high-risk sample *β* = 0.12, *P* = 0.007). We performed similar exclusions (low/moderate-risk sample *N* = 3, high-risk sample *N* = 48) for the further analysis predicting 2-h glucose (Table 4C), but this did not change the results.

## Discussion

This study examined the longitudinal relationships between chronic disease risk perceptions and behavioral and physiological disease risk indicators. We modeled how risk perceptions related to risk indicators together with self-efficacy and outcome beliefs, among different risk groups. People at high risk for diabetes clearly underestimated their risk: at baseline, the majority of the high-risk sample perceived their diabetes risk moderate, but a third of this sample were diagnosed with diabetes only 5 years later. Perceived risks of diabetes and CVD were not found to predict changes in physical activity, BMI, or blood glucose over 5 years. Instead, higher BMI and blood glucose tended to predict higher perceived disease risks. Higher self-efficacy predicted slightly increased physical activity among both samples. Higher outcome beliefs predicted slightly decreased BMI among the low/moderate-risk sample.

Over the 5-year follow-up, risk indicators tended to predict slightly increased risk perceptions, but not the reverse. The longitudinal associations were small, but the results rather support the accuracy hypothesis of risk perception than the behavioral motivation hypothesis [5]. Altogether, the results of this study suggest that perceived risk of chronic disease rather seems to reflect actual risk than predict health behavior change. Health examination–based physiological risk indicators, BMI and 2-h glucose, predicted quite systematically higher perceived risks of diabetes and CVD.

In contrast, self-reported physical activity showed a single weak longitudinal association with the unexpected direction. More frequent physical activity should, according to theory, predict lower perceived risk, when controlling for the baseline level of perceived risk. Weakness of the physical activity measure could explain why more frequent physical activity was not found to predict lower perceived risk: slightly different items were used at baseline and at follow-up, and incapability to exercise was only taken into account at follow-up. However, qualitative research suggests that people may not identify lack of physical activity as a risk factor to the same extent as experts do [26]. The current study results are in line with previous longitudinal studies where perceived risk was not found to predict physical activity [14].

A central limitation of this observational study was that it did not measure intention to change health behavior, which is a central concept in several health behavior theories. For example, the HAPA model expects that risk perception, self-efficacy, and outcome beliefs encourage intention to change behavior [13]. The HAPA model provides a conceptual framework for interpreting the current data, as the model includes the key predictors of this study, but we may not conclude whether risk perceptions were related to intentions, or whether intentions failed over 5 years. A recent meta-analysis of the HAPA model [16] found that self-efficacy and outcome beliefs had stronger effects on health behavior than perceived risk had. In the current study, higher self-efficacy predicted more frequent physical activity over 5 years, but no changes in BMI or blood glucose. Weak evidence was found for outcome beliefs predicting physical activity, BMI, or blood glucose. The observational study design may explain the weakness of these associations. Self-efficacy predicted successful weight management in several intervention studies [27]. In addition, the measure for outcome beliefs was a single item that concerned lifestyle in general, instead of physical activity or weight management specifically.

Risk communication is common in healthcare practice, but successful lifestyle change tends to require more than awareness of disease risks. Maintaining a high level of physical activity or losing weight permanently requires sustained efforts. Interventions designed to change diabetes risk perception have shown that even if risk feedback promotes more accurate risk perceptions, it may have no effect on health behavior or intentions [12]. This needs to be acknowledged when providing more and more individualized risk feedback, such as polygenic risk scores [28]. Intervention studies need to examine participant engagement and find the best ways to target risk perceptions together with other social cognitive factors [15, 29, 30]. Encouraging people to set goals and monitor their behavior seems to be a plausible strategy to promote healthy eating and physical activity [31].

The result that adults at high risk of diabetes underestimated their risk is in line with previous studies [32]. People tend to view their health and health behavior in favorable light [33]. A meta-analysis showed that optimistic bias in risk perception is highlighted when the risk concerns factors that are under one’s own control [34]. Physical activity is likely to be seen as more directly controllable than physiological measures, which could partly explain why physiological risk indicators predicted perceived risk more clearly than physical activity did. Even though participants’ risk perceptions were not accurate, disease risk perceptions did, to some degree, reflect actual risk indicators, as in previous research [20]. This is also in line with a cross-sectional study among the Finnish population, where risk perceptions of diabetes, CVD, cancer, and depression were related to family history and health behavior [35].

It should be noted, however, that in the current study, perceived risks were measured before participants received health examination–based feedback from several physiological measures, including blood glucose levels. Feedback on the health examination may have shifted risk perceptions and encouraged intentions to be physically active or lose weight [8], but these were not assessed in this study. When people receive information on multiple risk indicators, they seem to perceive their heightened risk relatively accurately [36]. However, people may also psychologically reject or minimize the personal relevance of the risk feedback [37], particularly if the feedback is negative, or inconsistent with one’s expectations [38]. Finally, at the time of the data collection of this study, between the years 2000 and 2010, there was a national diabetes prevention program in Finland [39]. The program aimed to promote diabetes risk awareness and lifestyle changes, and may have contributed to study participants’ risk perceptions.

### Strengths and Limitations

This study was able to examine three different chronic disease risk indicators and risk perception of two diseases in a longitudinal observational study design. Participants were derived from a nationally representative and comprehensive health study. However, it should be noted that this was a secondary analysis of an existing data set, and the data collection protocol was not originally designed for the purpose of the current investigation. Moreover, the original study was not designed to test the HAPA [13] or other health behavior models. As a result, perceived risks of diabetes and CVD were measured before and not right after the participants received health examination–based feedback, which may have shifted their risk perceptions. Risk perceptions can be quite resistant to change [40]. Although risk feedback may shift risk perceptions and motivate behavior change [8, 38], the evidence to date suggests that biomarker or polygenic risk feedback may not have a lasting impact on preventive health behaviors [12, 28].

Other limitations concern some of the self-reported measures. A self-reported single item was used for physical activity; this provides less precise information than more comprehensive measures or objective tools, such as accelerometers. Measures for self-efficacy and outcome beliefs were non-validated and concerned health behaviors in general, instead of weight management and physical activity, i.e., the study outcomes, more specifically. Since intentions were not measured, no conclusions can be drawn whether or not the feedback on the health examination contributed to intentions to change lifestyle, or whether such intentions were simply not accomplished over the years. Despite these limitations, combining three different risk indicators provided a broad picture on their relationship with perceived risk over several years. As the study included two samples of participants with a different diabetes risk status, it was able to show that the relationship of risk perception and risk indicators was similar among those with a high risk and among those with a lower risk.

## Conclusion

Individuals at high diabetes risk tend to underestimate their risk. Perceived risk of chronic disease rather follows risk indicators than predicts long-term health behavior changes. Risk perception alone is unlikely to predict sustained lifestyle changes. This needs to be kept in mind also in personalized medicine, which aims for individualized risk assessment and treatment. Future intervention studies need to target self-efficacy and examine the best ways to combine risk feedback with various behavior change techniques, to promote sustained lifestyle changes among people at high risk for chronic diseases [31, 42].

## References

[1] Lozano R, Naghavi M, Foreman K, Lim S, Shibuya K, Aboyans V, Abraham J, Adair T, Aggarwal R, Ahn SY, AlMazroa MA, Alvarado M, Anderson HR, Anderson LM, Andrews KG, Atkinson C, Baddour LM, Barker-Collo S, Bartels DH, Bell ML, Benjamin EJ, Bennett D, Bhalla K, Bikbov B, Abdulhak AB, Birbeck G, Blyth F, Bolliger I, Boufous S, Bucello C, Burch M, Burney P, Carapetis J, Chen H, Chou D, Chugh SS, Coffeng LE, Colan SD, Colquhoun S, Colson KE, Condon J, Connor MD, Cooper LT, Corriere M, Cortinovis M, de Vaccaro KC, Couser W, Cowie BC, Criqui MH, Cross M, Dabhadkar KC, Dahodwala N, de Leo D, Degenhardt L, Delossantos A, Denenberg J, Des Jarlais DC, Dharmaratne SD, Dorsey ER, Driscoll T, Duber H, Ebel B, Erwin PJ, Espindola P, Ezzati M, Feigin V, Flaxman AD, Forouzanfar MH, Fowkes FGR, Franklin R, Fransen M, Freeman MK, Gabriel SE, Gakidou E, Gaspari F, Gillum RF, Gonzalez-Medina D, Halasa YA, Haring D, Harrison JE, Havmoeller R, Hay RJ, Hoen B, Hotez PJ, Hoy D, Jacobsen KH, James SL, Jasrasaria R, Jayaraman S, Johns N, Karthikeyan G, Kassebaum N, Keren A, Khoo JP, Knowlton LM, Kobusingye O, Koranteng A, Krishnamurthi R, Lipnick M, Lipshultz SE, Ohno SL, Mabweijano J, MacIntyre MF, Mallinger L, March L, Marks GB, Marks R, Matsumori A, Matzopoulos R, Mayosi BM, McAnulty JH, McDermott MM, McGrath J, Memish ZA, Mensah GA, Merriman TR, Michaud C, Miller M, Miller TR, Mock C, Mocumbi AO, Mokdad AA, Moran A, Mulholland K, Nair MN, Naldi L, Narayan KMV, Nasseri K, Norman P, O’Donnell M, Omer SB, Ortblad K, Osborne R, Ozgediz D, Pahari B, Pandian JD, Rivero AP, Padilla RP, Perez-Ruiz F, Perico N, Phillips D, Pierce K, Pope CA, Porrini E, Pourmalek F, Raju M, Ranganathan D, Rehm JT, Rein DB, Remuzzi G, Rivara FP, Roberts T, de León FR, Rosenfeld LC, Rushton L, Sacco RL, Salomon JA, Sampson U, Sanman E, Schwebel DC, Segui-Gomez M, Shepard DS, Singh D, Singleton J, Sliwa K, Smith E, Steer A, Taylor JA, Thomas B, Tleyjeh IM, Towbin JA, Truelsen T, Undurraga EA, Venketasubramanian N, Vijayakumar L, Vos T, Wagner GR, Wang M, Wang W, Watt K, Weinstock MA, Weintraub R, Wilkinson JD, Woolf AD, Wulf S, Yeh PH, Yip P, Zabetian A, Zheng ZJ, Lopez AD, Murray CJL (2012). Global and regional mortality from 235 causes of death for 20 age groups in 1990 and 2010: a systematic analysis for the Global Burden of Disease Study 2010. Lancet.

[2] Ogurtsova K, da Rocha Fernandes JD, Huang Y, Linnenkamp U, Guariguata L, Cho NH, Cavan D, Shaw JE, Makaroff LE (2017). IDF diabetes atlas: global estimates for the prevalence of diabetes for 2015 and 2040. Diabetes Res Clin Pract.

[3] Lindström J, Ilanne-Parikka P, Peltonen M, Aunola S, Eriksson JG, Hemiö K, Hämäläinen H, Härkönen P, Keinänen-Kiukaanniemi S, Laakso M, Louheranta A, Mannelin M, Paturi M, Sundvall J, Valle TT, Uusitupa M, Tuomilehto J (2006). Sustained reduction in the incidence of type 2 diabetes by lifestyle intervention: follow-up of the Finnish Diabetes Prevention Study. Lancet.

[4] Schwarzer R (2008). Modeling health behavior change: how to predict and modify the adoption and maintenance of health behaviors. Appl Psychol.

[5] Weinstein ND, Nicolich M (1993). Correct and incorrect interpretations of correlations between risk perceptions and risk behaviors. Health Psychol.

[6] Sheridan SL, Viera AJ, Krantz MJ, Ice CL, Steinman LE, Peters KE, Kopin LA, Lungelow D, Cardiovascular Health Intervention Research and Translation Network Work Group on Global Coronary Heart Disease Risk (2010). The effect of giving global coronary risk information to adults: a systematic review. Arch Intern Med.

[7] Renner B, Schüz B, Sniehotta FF (2008). Preventive health behavior and adaptive accuracy of risk perceptions. Risk Anal.

[8] McClure JB (2002). Are biomarkers useful treatment aids for promoting health behavior change?: an empirical review. Am J Prev Med.

[9] French DP, Cameron E, Benton JS, Deaton C, Harvie M (2017). Can communicating personalised disease risk promote healthy behaviour change? A systematic review of systematic reviews. Ann Behav Med.

[10] Katsios C, Roukos DH (2010). Individual genomes and personalized medicine: life diversity and complexity. Pers Med.

[11] Plotnikoff RC, Trinh L (2010). Protection motivation theory: is this a worthwhile theory for physical activity promotion?. Exerc Sport Sci Rev.

[12] Godino JG, van Sluijs EM, Marteau TM, Sutton S, Sharp SJ, Griffin SJ (2016). Lifestyle advice combined with personalized estimates of genetic or phenotypic risk of type 2 diabetes, and objectively measured physical activity: a randomized controlled trial. PLoS Med.

[13] Schwarzer R, Schüz B, Ziegelmann JP, Lippke S, Luszczynska A, Scholz U (2007). Adoption and maintenance of four health behaviors: theory-guided longitudinal studies on dental flossing, seat belt use, dietary behavior, and physical activity. Ann Behav Med.

[14] Gholami M, Knoll N, Schwarzer R. Application of the health action process approach to physical activity: a meta-analysis. Self-Regul Health Behav Life Span. 2014;72.

[15] Sheeran P, Harris PR, Epton T (2014). Does heightening risk appraisals change people’s intentions and behavior? A meta-analysis of experimental studies. Psychol Bull.

[16] Zhang C-Q, Zhang R, Schwarzer R, Hagger MS. A meta-analysis of the health action process approach. Health Psychol. 2019; 10.1037/hea0000728 30973747

[17] Laatikainen T, Tapanainen H, Alfthan G, Salminen I, Sundvall J, Leiviskä A, et al. Tutkimus kroonisten kansantautien riskitekijöistä, niihin liittyvistä elintavoista, oireista, psykososiaalisista tekijöistä ja terveyspalvelujen käytöstä. Natl Public Health Inst Hels Finl B 2003;7.

[18] Lindström J, Tuomilehto J (2003). The diabetes risk score: a practical tool to predict type 2 diabetes risk. Diabetes Care.

[19] Alberti KGMM, Zimmet P ft. Definition, diagnosis and classification of diabetes mellitus and its complications. Part 1: diagnosis and classification of diabetes mellitus. Provisional report of a WHO consultation. Diabet Med 1998;15(7):539–553. 10.1002/(SICI)1096-9136(199807)15:7<539::AID-DIA668>3.0.CO;2-S 9686693

[20] Godino JG, van Sluijs EM, Sutton S, Griffin SJ (2014). Understanding perceived risk of type 2 diabetes in healthy middle-aged adults: a cross-sectional study of associations with modelled risk, clinical risk factors, and psychological factors. Diabetes Res Clin Pract.

[21] WHO. Obesity: preventing and managing the global epidemic [Internet]. World Health Organization; 2000 [cited 2015 Jun 29]. Available from: http://www.google.com/books?hl=fi&lr=&id=AvnqOsqv9doC&oi=fnd&pg=PA1&dq=Obesity:+Preventing+and+managing+the+global+epidemic.+Report+of+a+WHO+Consultation+(WHO+Technical+Report+Series+894).&ots=6UG59pUW5N&sig=vGrVRkyGPvkVG49nrJ4yOy1LrMk 11234459

[22] Borodulin K. Physical activity, fitness, abdominal obesity, and cardiovascular risk factors in Finnish men and women: the National FINRISK 2002 study Kansanterveyslaitoksen Julk A 2006;

[23] Shaw JE, Zimmet PZ, de Courten M, Dowse GK, Chitson P, Gareeboo H a (1999). Impaired fasting glucose or impaired glucose tolerance. What best predicts future diabetes in Mauritius?. Diabetes Care.

[24] Saaristo T, Moilanen L, Korpi-Hyövälti E, Vanhala M, Saltevo J, Niskanen L (2010). Lifestyle intervention for prevention of type 2 diabetes in primary health care: one-year follow-up of the Finnish National Diabetes Prevention Program (FIN-D2D). Diabetes Care.

[25] Hu L, Bentler PM (1999). Cutoff criteria for fit indexes in covariance structure analysis: conventional criteria versus new alternatives. Struct Equ Model Multidiscip J.

[26] Damman OC, Timmermans DR (2012). Educating health consumers about cardio-metabolic health risk: what can we learn from lay mental models of risk?. Patient Educ Couns.

[27] Teixeira PJ, Carraça EV, Marques MM, Rutter H, Oppert J-M, De Bourdeaudhuij I (2015). Successful behavior change in obesity interventions in adults: a systematic review of self-regulation mediators. BMC Med.

[28] Hollands GJ, French DP, Griffin SJ, Prevost AT, Sutton S, King S (2016). The impact of communicating genetic risks of disease on risk-reducing health behaviour: systematic review with meta-analysis. Bmj..

[29] Harvey JN, Lawson VL (2009). The importance of health belief models in determining self-care behaviour in diabetes. Diabet Med.

[30] Rimal RN (2001). Perceived risk and self-efficacy as motivators: understanding individuals’ long-term use of health information. J Commun.

[31] Michie S, Abraham C, Whittington C, McAteer J, Gupta S (2009). Effective techniques in healthy eating and physical activity interventions: a meta-regression. Health Psychol.

[32] Adriaanse MC, Twisk JW, Dekker JM, Spijkerman AM, Nijpels G, Heine RJ (2008). Perceptions of risk in adults with a low or high risk profile of developing type 2 diabetes; a cross-sectional population-based study. Patient Educ Couns.

[33] Rothman A, Kiviniemi MT. Treating people with information: an analysis and review of approaches to communicating health risk information. 1999 [cited 2015 Oct 2]; Available from: http://digitalcommons.unl.edu/psychfacpub/14/10.1093/oxfordjournals.jncimonographs.a024207 10854457

[34] Klein CT, Helweg-Larsen M (2002). Perceived control and the optimistic bias: a meta-analytic review. Psychol Health.

[35] Vornanen M, Konttinen H, Kääriäinen H, Männistö S, Salomaa V, Perola M, Haukkala A (2016). Family history and perceived risk of diabetes, cardiovascular disease, cancer, and depression. Prev Med.

[36] Gamp M, Schupp HT, Renner B. Risk perceptions after receiving multiple risk feedback. Personal Soc Psychol Bull. 2018:0146167218767877. 10.1177/0146167218767877 29716423

[37] Vähäsarja K, Kasila K, Kettunen T, Rintala P, Salmela S, Poskiparta M (2015). ‘I saw what the future direction would be…’: experiences of diabetes risk and physical activity after diabetes screening. Br J Health Psychol.

[38] Renner B (2004). Biased reasoning: adaptive responses to health risk feedback. Personal Soc Psychol Bull.

[39] Saaristo T, Peltonen M, Keinänen-Kiukaanniemi S, Vanhala M, Saltevo J, Niskanen L, Oksa H, Korpi-Hyøvälti E, Tuomilehto J (2007). National type 2 diabetes prevention programme in Finland: FIN-D2D. Int J Circumpolar Health.

[40] Wang C, Sen A, Ruffin MT, Nease DE, Gramling R, Acheson LS (2012). Family history assessment: impact on disease risk perceptions. Am J Prev Med.

[41] WMA. WMA Declaration of Helsinki - ethical principles for medical research involving human subjects [Internet]. 2013 [cited 2017 Mar 21]. Available from: http://www.wma.net/en/30publications/10policies/b3/index.html

[42] Reiner Z, Sonicki Z, Tedeschi-Reiner E. Public perceptions of cardiovascular risk factors in Croatia: the PERCRO survey. Reiner Z, Sonicki Z, Tedeschi-Reiner E. Prev Med. 2010 Dec;51(6):494–6). 10.1016/j.ypmed.2010.09.015 20951724

